# Urolithin A Modulates PER2 Degradation via SIRT1 and Enhances the Amplitude of Circadian Clocks in Human Senescent Cells

**DOI:** 10.3390/nu17010020

**Published:** 2024-12-25

**Authors:** Rassul Kuatov, Jiro Takano, Hideyuki Arie, Masaru Kominami, Norifumi Tateishi, Ken-ichi Wakabayashi, Daisuke Takemoto, Takayuki Izumo, Yoshihiro Nakao, Wataru Nakamura, Kazuyuki Shinohara, Yasukazu Nakahata

**Affiliations:** 1 Department of Neurobiology & Behavior, Graduate School of Biomedical Sciences, Nagasaki University, Nagasaki 852-8523, Japan; 2 Institute for Science of Life, Suntory Wellness Limited, Kyoto 619-0284, Japannorifumi_tateishi@suntory.co.jp (N.T.); takayuki_izumo@suntory.co.jp (T.I.);; 3 Department of Oral-Chrono Physiology, Graduate School of Biomedical Sciences, Nagasaki University, Nagasaki 852-8588, Japan

**Keywords:** circadian clock, senescent cells, urolithin A, ellagic acid, antiaging

## Abstract

Background/Objectives: Circadian clocks are endogenous systems that regulate numerous biological, physiological, and behavioral events in living organisms. Aging attenuates the precision and robustness of circadian clocks, leading to prolonged and dampened circadian gene oscillation rhythms and amplitudes. This study investigated the effects of food-derived polyphenols such as ellagic acid and its metabolites (urolithin A, B, and C) on the aging clock at the cellular level using senescent human fibroblast cells, TIG-3 cells. Methods: Lentivirus-infected TIG-3 cells expressing Bmal1-luciferase were used for real-time luciferase monitoring assays. Results: We revealed that urolithins boosted the amplitude of circadian gene oscillations at different potentials; urolithin A (UA) amplified the best. Furthermore, we discovered that UA unstabilizes PER2 protein while stabilizing SIRT1 protein, which provably enhances *BMAL1* oscillation. Conclusions: The findings suggest that urolithins, particularly UA, have the potential to modulate the aging clock and may serve as therapeutic nutraceuticals for age-related disorders associated with circadian dysfunction.

## 1. Introduction

In almost all living organisms on Earth, numerous biological, physiological, and behavioral events exhibit oscillations of approximately 24 h. They have evolutionarily developed an endogenous system called “the circadian clock” to adapt to Earth’s rotation with similar regulatory mechanisms. Light exposure, physical activity, and eating habits can influence and reset our internal circadian clock [1,2]. Circadian molecular oscillators consist of positive and negative elements that form transcription/translation-based feedback loops (TTFLs) [1,3,4]. The TTFLs are negative feedback loops, in which clock genes are regulated by their protein products. The mammalian circadian oscillator is composed of positive regulators (CLOCK, BMAL1, and ROR) and negative regulators (PERIODs (PERs), CRYPTOCHROMEs (CRYs), and REV-ERBs). BMAL1 forms heterodimers with CLOCK, and *PERs* and *CRYs* are target genes of CLOCK/BMAL1. The encoded proteins, PERs and CRYs, form a complex to repress CLOCK/BMAL1-dependent transcription, which provides negative feedback. This core feedback loop is coupled to an interlocked loop, in which the nuclear receptors RORs and REV-ERBs form an additional feedback loop. RORs and REV-ERBs competingly bind to the element RORE (ROR/REV-ERB-response element) in a time-dependent manner to form additional TTFLs to impinge on the core clock feedback loop.

The main center of the circadian clock, referred to as the central or master clock, is located in the suprachiasmatic nucleus (SCN) of the hypothalamus [3,4]. On the other hand, almost all cells in the body, including primary and cultured cells, also possess circadian clocks called peripheral clocks [2,3,4]. Recent studies have revealed that 10–30% of genes expressed in tissues are clock-controlled [5,6], supporting that a wide array of biological and physiological events is regulated by the circadian clock. Of note, even in chronic pathological conditions like rheumatoid arthritis, the circadian clock modulates the biochemistry, immune/inflammatory reactivity, and related symptoms, such as morning stiffness, joint pain, and swelling, in accordance with circadian and circannual rhythms [7,8]. Both central and peripheral clocks are precise and robust; however, it has been reported that aging attenuates the precision and robustness of the circadian clock, hereafter referred to as the aging clock [9,10,11,12,13,14,15]. At the animal level, the free-running period increases, and the timing of the activity onset shows more variation among days with aging [9,11]. At the tissue level, PER2::LUC rhythms in the SCN of aged animals showed markedly lower amplitudes and longer circadian periods than those of young animals [10]. We recently reported that even at the cellular level, the circadian gene oscillation rhythm and amplitude are prolonged and dampened, respectively, with senescence [16,17]. This led to the idea of using senescent cells to discover natural or chemical compounds that can overcome the aging clock [18].

Ellagic acid (EA) is a polyphenol found in a variety of plants, including nuts, grapes, pomegranates, berries, fruits, and seeds such as pecans [19,20,21]. Characterized by hydrophobic moiety contributing to its low water solubility, EA is only partially absorbed in the small intestine [22]. Further, it is metabolized by the microbiota of the large intestine into a class of compounds known as urolithins (Urolithin A-D). Possessing anti-tumor, anti-oxidation, anti-inflammation, neuroprotective, and anti-aging effects, EA and its metabolites (especially urolithin A) are of significant interest to researchers worldwide [23,24,25,26,27]. EA and some of its metabolites can inhibit key inflammation signaling pathways, such as NF-κB, MAPK, and JAK-STAT, mitigating chronic inflammation that contributes to aging [28,29].

The effects of EA and urolithin A on the circadian clock have recently been reported using mouse embryonic fibroblasts, intestinal epithelium cells, and young and old mice [30,31]. However, the effects of EA and its metabolites on the circadian clock in senescent cells remain unclear. In this study, we investigated the effects of EA and its metabolites on the circadian clock in human senescent cells. We revealed that the EA metabolites, urolithin A, B, and C, amplify the amplitude of the circadian clock. Furthermore, we demonstrated that PER2 was unstabilized by urolithin A, suggesting the molecular mechanisms by which urolithin A amplifies circadian gene oscillations.

## 2. Materials and Methods

### 2.1. Reagents

Ellagic acid (EA) was purchased from Tokyo Chemical Industry Co., LTD (E0375), and urolithin A (UA), urolithin B (UB), and urolithin C (UC) were purchased from Toronto Research Chemicals Inc. (TRC-U847000, TRC-U847005, and TRC-U847015).

### 2.2. Cell Culture

We used human fetal lung-derived primary diploid fibroblasts, TIG-3 cells, and proliferative and senescent TIG-3 cells used in this study were established in our previous report [16], which consisted of cells in the passage range of P25-29 and P36-41, respectively. Cells used in this study were cultured in DMEM-4.5 g/L glucose (Nacalai Tesque, Kyoto, Japan) supplemented with 10% FBS (Sigma-Aldrich, St. Louis, MO, USA) and antibiotics (100 units/mL penicillin, 100 µg/mL streptomycin, Nacalai Tesque, Japan) at 37 °C and 5% CO_2_ in a humidified incubator.

### 2.3. Lentivirus Production, Infection to TIG-3 Cells, and Real-Time Luciferase Monitoring Assay

Lentivirus production, infection to TIG-3 cells, and real-time luciferase monitoring assays were performed as previously described [16], with minor modifications. Briefly, HEK293T cells were seeded at 3 × 10^6^ cells in a 10 cm dish. After 24 h of seeding, 3 μg of psPAX2, 2 μg of pMD2.G, and 5 μg of pLV6-Bmal1-luc vectors were transfected with 20 μL of 1 mg/mL polyethylenimine (PEI; Polysciences, Warrington, PA, USA). After 24 h, the medium was replaced with 5 mL fresh culture medium. After 24 h, the viral supernatant was collected and stored at 4 °C, and 5 mL of fresh culture medium was added to the dish again. After 24 h, the viral supernatant was collected and pooled with the viral supernatant from the previous day. Cellular debris in the viral supernatant was removed by centrifugation and 0.2 μm filter filtration. The filtered viral suspension was then centrifuged at 8000× *g* at 4 °C for >4 h. Then, the lentivirus pellet was resuspended in 2 mL of medium.

The culture medium for TIG-3 cells, seeded the previous day at 1.5 × 10^5^ cells/35 mm dish, was replaced with virus suspension for 24 h. The cells were then incubated for an additional 48 h before performing the real-time luciferase monitoring assay.

Infected TIG-3 cells were treated with 0.1 μM dexamethasone (Dex; Nacalai Tesque, Japan) for 1 h to synchronize the circadian clocks, then the cells with the culture medium containing 100 μM D-luciferin (Nacalai Tesque, Japan) were placed in the KronosDio luminometer (ATTO, Tokyo, Japan) and the bioluminescence of the cells was measured for 5 days continuously.

### 2.4. Analyses of the Circadian Period and Amplitude

We performed two analytical methods for evaluating the circadian rhythm, the cosinor and manual analyses. To perform the cosinor analysis, detrended data from 20-68 h were used. Cosinor software version 3.1 was kindly provided by Dr. R Refinetti [32]. The method for the manual analysis is shown in Supplementary Figure S1.

### 2.5. RNA Extraction and qPCR

RNA extraction and qPCR were performed as previously described [33]. Briefly, total RNA from TIG-3 cells was extracted using Sepasol RNA-I Super G (Nacalai Tesque, Japan) and was reverse-transcribed using SuperScript II Reverse Transcriptase (Invitrogen, Waltham, MA, USA) with random primers. Quantitative PCR was performed in the presence of KAPA SYBR FAST Universal 2X qPCR Master Mix (Nippon Genetics, Japan) on a Light Cycler 480 (Roche, Basel, Switzerland) under the following conditions: 95 °C for 3 min, followed by 40 cycles at 95 °C for 10 s, 60 °C for 20 s, and 72 °C for 1 s. The primer sets for *hPER1*, *hPER2*, *hCRY1*, *hCRY2*, *hREV-ERBA*, *hREV-ERBB*, and *18S rRNA* have been previously described [16].

### 2.6. Quantification of Intracellular NAD^+^

Senescent TIG-3 cells were treated with the indicated reagents at the indicated concentrations for 24 h. NAD^+^ was measured using the NAD/NADH-Glo assay kit (Promega, Madison, WI, USA) according to the manufacturer’s instructions.

### 2.7. Protein Half-Life Assay

Full-length coding sequences without the stop codon of *mPer1*, *mPer2*, *mCry1*, *mRev-erbα*, or *mRev-erbβ* were subcloned into the pGL3 vector, which were inflamed with a luciferase coding sequence. These vectors were transfected into senescent TIG-3 cells by using FuGeneHD (Promega, USA). Twenty-four hours after transfection, cells with a culture medium containing 100 μg/mL cycloheximide and 100 μM D-luciferin were placed in the KronosDio luminometer, and the bioluminescence of the cells was measured continuously for 12 h.

### 2.8. SDS-PAGE and Western Blotting Analysis

The protocols for SDS-PAGE and western blot analysis were performed as described previously [33]. Protein measurements were performed using 660 Protein Assay Reagent (Thermo Fisher Scientific, Waltham, MA, USA) according to the manufacturer’s instructions. Antibodies against SIRT1 (07-131) andα-tubulin (T6074; clone B-5-1-2) were purchased from Millipore (Burlington, MA, USA) and Sigma-Aldrich, respectively.

### 2.9. Statistics

Values are presented as the mean ± SEM. The Student’s two-tailed *t*-test was applied for the comparisons of two datasets, and one-way analysis of variance (ANOVA) followed by Dunnett’s post-hoc test was analyzed by GraphPad Prism 10.3.0 applied to compare more than two groups. Statistical significance is indicated as * *p* < 0.05, ** *p* < 0.01, or *** *p* < 0.001.

## 3. Results

### 3.1. Urolithin A Boosted the Amplitude of Bmal1 Promoter-Driven Luciferase Oscillation

**Figure 1:**
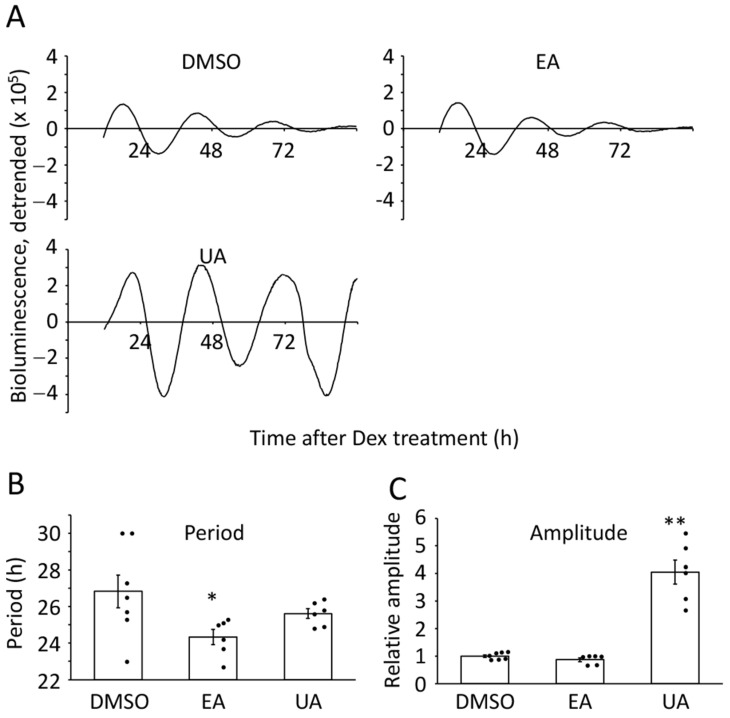
Effects of ellagic acid and urolithin A on the circadian clock of senescent cells. (**A**) Representative circadian oscillation patterns of DMSO-, ellagic acid (EA; 33.1 μM)-, or urolithin A (UA; 13.1 μM)-treated luciferase driven by *Bmal1* promoter were shown. (**B**,**C**) The period lengths and relative amplitudes were analyzed by the cosinor method using the data from (**A**). Each sample number was 6 or 7. The value of DMSO was set to 1 for the relative amplitude. ANOVA followed by Dunnett’s post-hoc test was analyzed. Statistical significance compared with the control “DMSO” is indicated as * *p* < 0.05, or ** *p* < 0.005.

To reveal the effects of food-derived polyphenols on circadian clock gene expression patterns in senescent cells, we prepared replicative senescent human fetal lung-derived diploid fibroblasts, TIG-3 cells, which were defined in our previous study [16]. Senescent cells expressing the *Bmal1* promoter-driven luciferase gene were treated with vehicle (0.5% DMSO), EA (33.1 μM ellagic acid), or UA (13.1 μM urolithin A) after the synchronization of the circadian clock in cells by dexamethasone treatment (Figure 1A). To prove the period length and amplitude, we analyzed the data by the cosinor method [32] (Figure 1B,C) and manually (Supplementary Figure S1). Taken from both analyses, we concluded that UA enhanced the amplitude by approximately 4-fold but had no effect on the circadian period, and EA had no impact on both circadian properties.

**Figure 2:**
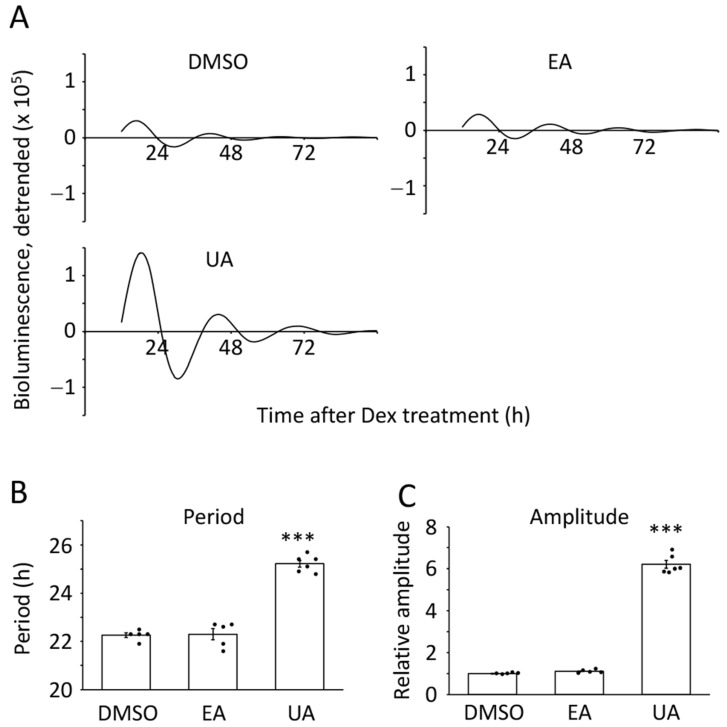
Effects of ellagic acid and urolithin A on the circadian clock of proliferative cells. (**A**) Representative circadian oscillation patterns of DMSO-, EA (33.1 μM)-, or UA (30 μM)-treated luciferase driven by *Bmal1* promoter were shown. (**B**,**C**) The period lengths and relative amplitudes were analyzed by the cosinor method using the data from (**A**). Each sample number was 5 or 6. The value of DMSO was set to 1 for the relative amplitude. ANOVA followed by Dunnett’s post-hoc test was analyzed. Statistical significance compared with the control “DMSO” is indicated as *** *p* < 0.001.

To confirm whether the effects of EA and UA are specific to the senescent cells or more general to the human cells, we next performed the same experiments using the proliferative TIG-3 cells, which were also defined in our previous study [16]. As shown in Figure 2, EA had no effects on the period and amplitude, which are the same as those in the senescent cells. UA also had the same effect on the amplitude; however, it had a different impact on the period length. UA prolonged it, which is consistent with the result of MEFs reported by Haraguchi et al. [31]. Manual analysis also showed the same results (Supplementary Figure S2). The experiments so far indicate that EA has no effects on the circadian clock in human cells in vitro; however, UA has impacts on the period of the proliferative cells and the amplitude of both proliferative and senescent cells.

### 3.2. Ellagic Acid-Derived Metabolites Boosted the Amplitude of Bmal1 Promoter-Driven Luciferase Oscillation in Senescent Cells

**Figure 3:**
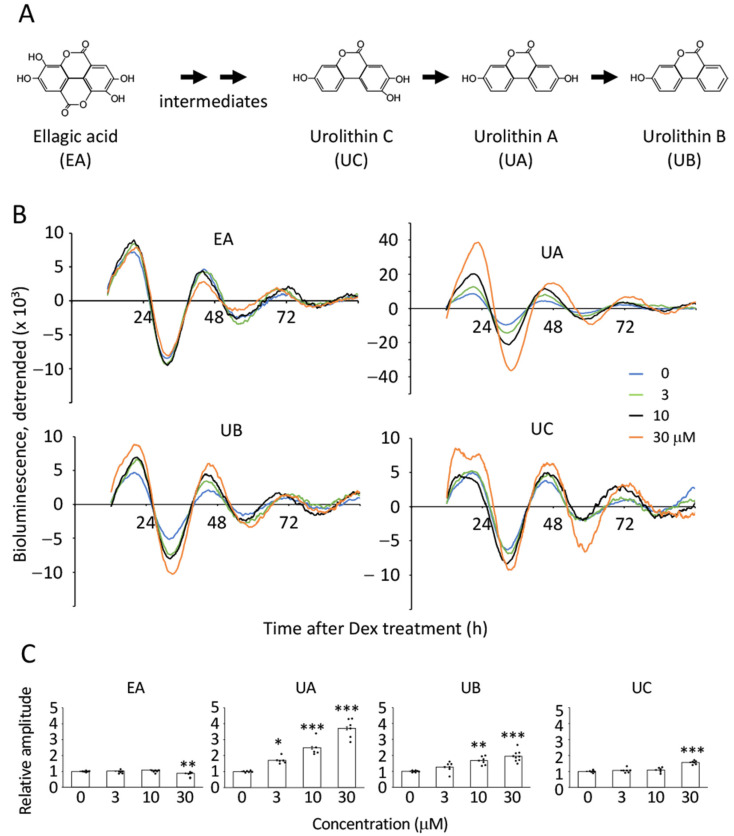
Effects of EA and its derivatives on the circadian clock of senescent cells. (**A**) Ellagic acid and its metabolites are shown. (**B**) Representative circadian oscillation patterns of EA-, UA-, UB, or UC-treated luciferase driven by *Bmal1* promoter were shown. (**C**) Amplitudes were analyzed with the cosinor method using the data from (**B**), and the amplitude of 0 mM for each metabolite was set to 1. Each sample number was 5 to 8. Values are presented as the mean ± SEM. ANOVA followed by Dunnett’s post-hoc test was analyzed. Statistical significance compared with the control “0” is indicated as * *p* < 0.05, ** *p* < 0.01, or *** *p* < 0.001.

As UA enhanced the amplitude of the *Bmal1* promoter-driven luciferase oscillation in TIG-3 cells irrespective of whether they were proliferating or senescent cells, we next assessed whether other ellagic acid-derived metabolites, urolithin B and C [34,35] (Figure 3A), have the potential to enhance amplitude in senescent cells. The senescent cells were treated with EA, UA, UB, or UC at the indicated concentrations after dexamethasone treatment (Figure 3B). As shown in Figure 1, EA had no effect or slightly decreased amplitude, and UA demonstrated enhanced amplitudes in a dose-dependent manner (Figure 3B,C). UB showed a dose-dependent amplitude enhancement; however, it was smaller than that of UA. On the other hand, UC enhanced the amplitude only at the highest concentration, with the smallest changes (Figure 3B,C). Manual analysis also showed the same tendency (Supplementary Figure S3A). Again, we could not find any effects of EA and its metabolites on the circadian period (Supplementary Figure S3B). Taken together, we revealed that EA metabolites, but not EA itself, possess the potential to enhance the circadian amplitude. Furthermore, among the EA metabolites, UA had the strongest effect on amplitude enhancement without changing the period length.

**Figure 4:**
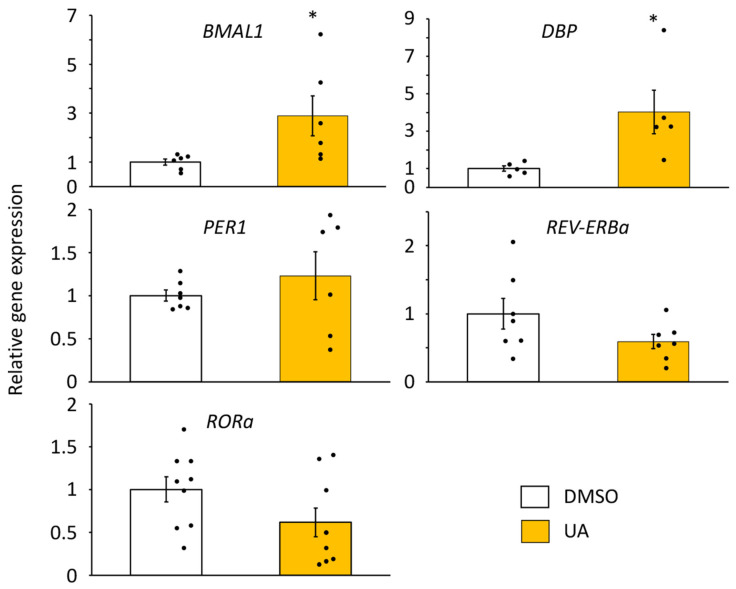
Effects of UA on the circadian clock gene expressions in senescent cells. Circadian gene expression levels after the UA treatment were quantified by qPCR. Each sample was normalized by the amount of *18S rRNA*. Each gene expression level in DMSO was set to 1. Each sample number was 5 to 9. Values are presented as the mean ± SEM. The Student’s two-tailed *t*-tests were performed. Statistical significance compared with the control “DMSO” is indicated as * *p* < 0.05.

We further investigated whether the expression levels of endogenous clock genes in senescent cells were altered by UA treatment. UA treatment increased *BMAL1* mRNA levels (Figure 4), consistent with the results shown in Figure 1 and Figure 3, in which we used *Bmal1* promoter-driven luciferase. We also analyzed other core circadian genes and found that *DBP* mRNA was increased; however, *PER1*, *RORa*, and *REV-ERBa* mRNA levels were comparable to those of DMSO-treated samples (Figure 4).

### 3.3. UA Treatment Unstabilized Per2-luc Fusion Protein

**Figure 5:**
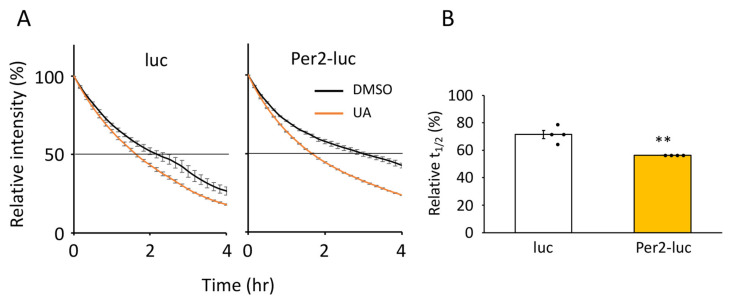
Effects of UA on Per2 protein stability in senescent cells. (**A**) The protein stability of luciferase-fused Per2 protein (Per2-luc) or luciferase alone (luc) was measured using the real-time monitoring system. (**B**) Effects of UA on protein stability were analyzed. Values indicate the percentages of t_1/2_ of the UA-treated condition divided by t_1/2_ of the DMSO-treated condition. Each sample number was 4. Values are presented as the mean ± SEM. The Student’s two-tailed *t*-tests were performed. Statistical significance compared with the control “luc” is indicated as ** *p* < 0.01.

Next, we investigated the molecular mechanisms by which *Bmal1* amplitude is enhanced in UA-treated senescent cells. Using a luciferase fusion protein assay system, we first investigated whether UA treatment altered the stability of clock proteins. C-terminally luciferase-fused clock proteins were expressed in senescent TIG-3 cells, and luciferase intensities were monitored after cycloheximide treatment using a real-time monitoring system. The screening revealed that UA treatment changed only the stability of Per2-luc among the five fusion proteins (Supplementary Figure S4). Therefore, we focused on Per2-luc protein stability and found that Per2-luc was unstabilized by UA treatment (Figure 5A). The half-lives of Per2-luc were 1.7 ± 0.0 h under UA-treated condition and 3.0 ± 0.2 h under DMSO-treated condition (*p* = 6.0 × 10^−4^). Since luc alone was also unstabilized by UA treatment (UA; 1.7 ± 0.1 h vs. DMSO; 2.3 ± 0.2 h, *p* = 0.01), we further analyzed UA’s effects on times reaching intensity to 50% (t_1/2_) (Figure 5B). Compared with the t_1/2_ by DMSO treatment, the t_1/2_ by UA treatment were 56.3 ± 8.4% and 71.4 ± 2.9% for Per2-luc and luc alone (*p* = 0.002), respectively. These data indicate that UA promoted the degradation of Per2 protein.

### 3.4. SIRT1 Was Upregulated in UA-Treated Senescent Cells

**Figure 6:**
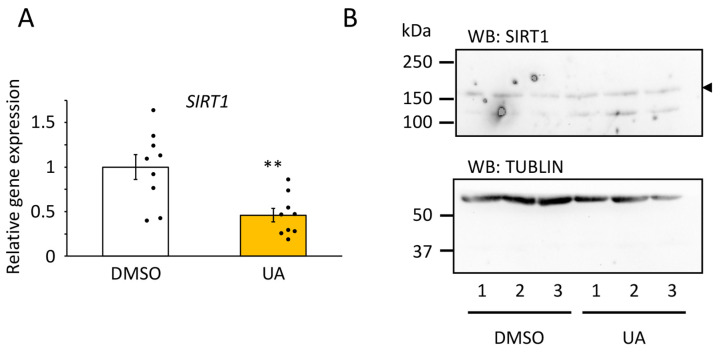
Effects of UA on Sirt1 amount in senescent cells. (**A**) *SIRT1* expression levels after the UA treatment were quantified by qPCR. Samples were normalized by the amount of *18S rRNA*. *SIRT1* expression level in DMSO was set to 1. The sample numbers were 9. Values are presented as the mean ± SEM. The Student’s two-tailed *t*-tests were performed. Statistical significance compared with the control “DMSO” is indicated as ** *p* < 0.01. (**B**) SIRT1 (upper panel) and a-TUBLIN (bottom panel) protein levels under indicated conditions were detected. The arrowhead indicates non-specific bands.

**Figure 7:**
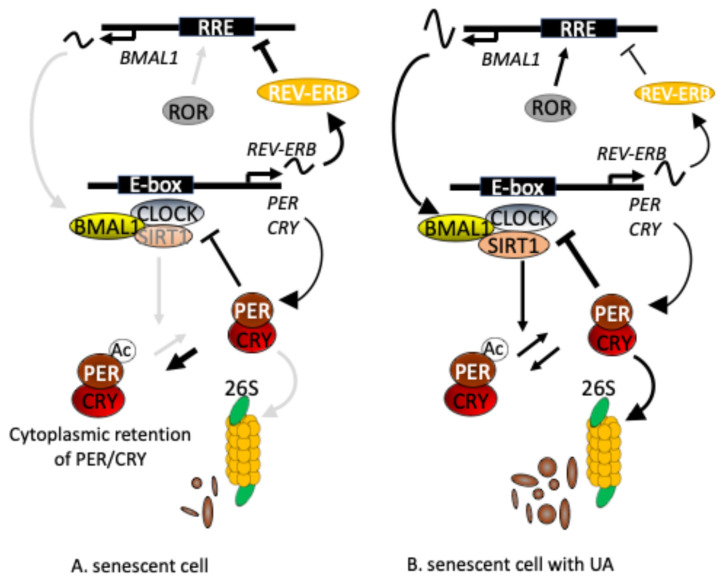
Scheme of how UA amplifies circadian gene expression in senescent cells. UA treatment increases the SIRT1 protein amount, which may promote its deacetylation activity and subsequent degradation of PER2, thereby releasing CLOCK/BMAL1 repression by PER/CRY. This enhances oscillatory *REV-ERB* and thereby *BMAL1* gene expression.

Per2 protein stability is partially regulated by its acetylation status; deacetylation of Per2 by SIRT1 promotes its degradation via the ubiquitin-proteasome system [36]. Since SIRT1 is an NAD^+^-dependent deacetylase [37], we first assessed whether UA increased intracellular NAD^+^ levels in senescent TIG-3 cells. FK866, an inhibitor of NAMPT, the rate-limiting enzyme of the NAD^+^ salvage pathway, and nicotinamide adenine mononucleotide (NMN), the precursor of NAD^+^, decreased and increased intracellular NAD^+^, respectively, as reported previously [38], whereas neither EA nor UA altered intracellular NAD^+^ levels (Supplementary Figure S5). We next investigated whether the levels of *SIRT1* mRNA and protein were increased by UA treatment in senescent cells and found that SIRT1 protein levels tended to be increased under UA-treated conditions (Figure 6B). However, *SIRT1* mRNA levels were decreased by 50% under UA-treated conditions (Figure 6A). These results imply the following steps (Figure 7): (1) stabilization of SIRT1 protein by UA treatment increases its deacetylation potential, which promotes deacetylation of PER and the subsequent nuclear entry of PER/CRY to repress CLOCK/BMAL1 activity [38], and degradation of PER2 (Figure 5) [36], thus CLOCK/BMAL1 has robust rhythmic activity; then, (2) the robust rhythmic CLOCK/BMAL1 transcription induces more rhythmic transcriptional regulation of *REV-ERB*, which in turn creates *BMAL1* robust rhythmic expression.

## 4. Discussion

Aging has garnered attention as a risk factor linked to alterations in circadian behavior and physiological/biochemical events from the animal to the cellular level. At the animal level, free-running periods and phase angles are altered with aging. We recently reported that the senescent cells possess a prolonged circadian period with attenuated amplitude [16,17], which was also observed in aged versus young mouse SCN ex vivo PER2::LUC monitoring [10]. Intriguingly, perturbations of the circadian clock demonstrate the acceleration of aging, thereby indicating that aging and circadian clocks have mutual regulation. However, it remains unclear whether recuperating the circadian clock slows the aging process. With aging, the amplitude of circadian rhythms generally decreases, which can be observed in various measures like waking activity, body temperature, SCN firing, and hormone levels [14,15]. Our study demonstrated that the EA metabolites, UA, UB, and UC, enhanced the amplitude of circadian gene expression in senescent cells; UA possessed the greatest potential for amplitude enhancement, which might increase the amplitude of many physiological events regulated by the circadian clock in vivo, especially for the middle or elder age in low UA population (see below). Our study demonstrated that the EA metabolites, UA, UB, and UC, enhanced the amplitude of circadian gene expression; UA possessed the greatest potential for amplitude enhancement (Figure 3). Studies of the effects of EA and UA on the circadian clock have been reported recently [30,31], showing the possibility that EA and UA are novel modulators of the circadian clock in vitro and in vivo; Haraguchi et al. investigated the effects of EA and UA on MEFs and young/old mice. At the cellular level, consistent with our finding (Figure 2), UA prolonged the circadian period and enhanced the amplitude [31]. Aged mice fed a diet containing oak extract, which is metabolized into EA and urolithins, increased the daily activity/rest ratio, which was not observed in young mice. Moreover, young mice fed an oak extract diet did not prolong the circadian period under constant dark conditions. Although further extensive studies will be required to assess the effects of UA on the circadian rhythm and overall health in humans, randomized clinical trials have recently shown that a daily UA supplementation of 1000 mg is safe and profitable for the older population [39,40].

Urolithins are natural compounds metabolized by the gut microbiome from EA (Figure 3A), a polyphenol abundant in foods such as pomegranates, berries, and nuts [34,41]. UA has been shown to reduce intestinal inflammation and enhance intestinal barrier function [42]. Du et al. recently reported that UA improved the rhythmic gene expressions of the circadian clock and tight junctions in intestinal cells in vitro and in vivo [30]. Remarkably, according to EA metabolism, metabotypes are classified into three groups: individuals producing only UA as metabotype A, UM-A, UA, iso-UA, and UB as metabotype B, UM-B, and unable to produce any forms of urolithin as metabotype 0, UM-0 [43]. The percentage of individuals with UM-0 remains constant at approximately 10% from childhood to old age. In childhood, 80% of the population is UM-A; however, it begins to decline in the late teens and decreases to 55% in those over 40 years of age, which is concomitant with an increase of UM-B up to 45% from 10%. The shift of UM-A to UM-B by age 40 in a third of the total population might lead to attenuated circadian amplitude, increased intestinal inflammation, and weakened intestinal barrier. Therefore, UA may be a potent postbiotic to combat aging by modulating the circadian clock in vivo.

Urolithins are known to possess antioxidative potency, which has been reported to be correlated with the number of OH groups and lipophilicity of the molecules [44]. They showed that UC had the highest antioxidant activity (IC_50_ = 0.16 μM) and UA had less activity (IC_50_ = 13.6 μM), while UB did not show any antioxidant activity. The potency of circadian amplitude is UA > UB > UC; this order is not consistent with antioxidative potency; therefore, changes in cellular redox status by urolithins might not be associated with circadian amplification.

We previously reported that intracellular NAD^+^ levels decrease in senescent cells and modulate the amplitude of the circadian rhythm; low NAD^+^ levels attenuate the amplitude via PER2 cytosolic retention, probably acetylated PER2, due to low SIRT1 deacetylation activity [38]. Several studies have reported that UA increases intracellular NAD^+^ and/or SIRT1 levels [45,46,47]. However, UA-treated senescent TIG-3 cells showed similar intracellular NAD^+^ levels to non-treated senescent cells (Supplementary Figure S5), whereas SIRT1 protein amounts increased following UA treatment in senescent cells (Figure 6). SIRT1 deacetylates PER2, leading to its degradation through the ubiquitin-proteasome system [36]. Our findings imply that UA treatment increases the amount of SIRT1 and its deacetylation potential, leading to nuclear localization [38], and degradation of PER2 (Figure 5) [36], which induces robust rhythmic CLOCK/BMAL1 transcriptional activity, enhancing REV-ERB oscillatory expression followed by enhanced BMAL1 amplitude (Figure 7). On the other hand, UA inhibits multiple signaling pathways, such as NF-κB, MAPK, and JAK-STAT [28,29], which are known to be associated with cellular senescence as well as the circadian clock [18]. Guo et al. recently showed that UA may regulate the Hippo/YAP1 signaling pathway [48], and interestingly, Azzi et al. reported that YAP1 positively regulates the expression of CRY1, leading to fine-tuning of the pathway by adjusting the activation of YAP1 [49]. These reports suggest that UA may modulate the circadian clock in senescent cells via complex pathways. Further study will digest the molecular mechanisms between UA and the circadian clock in senescent cells.

As this study identified candidate compounds that restore the aging clock in vitro, it is crucial to further investigate whether UA administration could restore the aging clock in vivo and recuperate aging in animals and humans. Finally, we propose that the assay used in this study is a convenient and useful system for screening/discovering natural or chemical compounds that recuperate the aging clock in vitro.

## 5. Conclusions

In conclusion, this study demonstrates that urolithin A (UA), a metabolite of ellagic acid (EA), has the potential to modulate the circadian clock in senescent human cells. Our findings indicate that UA can partially restore the dampened circadian rhythms characteristic of senescent cells. Given the important role of circadian clocks in regulating many physiological processes, UA supplementation could potentially help mitigate some age-related declines in circadian function. However, further research is needed to determine whether these in vitro effects translate to meaningful benefits in vivo. This study also demonstrates the utility of senescent cell models for screening compounds that can modulate the aging clock. The assay system developed here could be valuable for identifying other natural or synthetic molecules capable of restoring robust circadian rhythms in senescent cells. Overall, these results provide new insights into the effects of urolithins on cellular circadian clocks and suggest UA as a promising compound for further investigation as a potential “chronotherapeutic” to combat age-related circadian dysfunction. Future studies examining the effects of UA on circadian rhythms in animal models and human subjects will be important next steps in evaluating its therapeutic potential.

## Data Availability

The original contributions presented in this study are included in the article/supplementary material. Further inquiries can be directed to the corresponding author.

## Supplementary Materials

The following supporting information can be downloaded at: https://www.mdpi.com/article/10.3390/nu17010020/s1. Figure S1: Effects of ellagic acid and urolithin A on the circadian clock of senescent cells; Figure S2: Effects of ellagic acid and urolithin A on the circadian clock of proliferative cells; Figure S3: Effects of EA and its derivatives on the circadian clock of senescent cells; Figure S4: Effects of UA on the stability of circadian clock proteins in senescent cells; Figure S5: Effects of UA on NAD+ amount in senescent cells.
