# Supplementary material for: Urolithin A Modulates PER2 Degradation via SIRT1 and Enhances the Amplitude of Circadian Clocks in Human Senescent Cells
Source: Nutrients. 2024 Dec 25;17(1):20. doi: 10.3390/nu17010020 (PMC11722880; doi:10.3390/nu17010020)
Supplement: Supplementary file 1 [file nutrients-17-00020-s001.zip › SFig.1_R1.pdf]

SFig.1

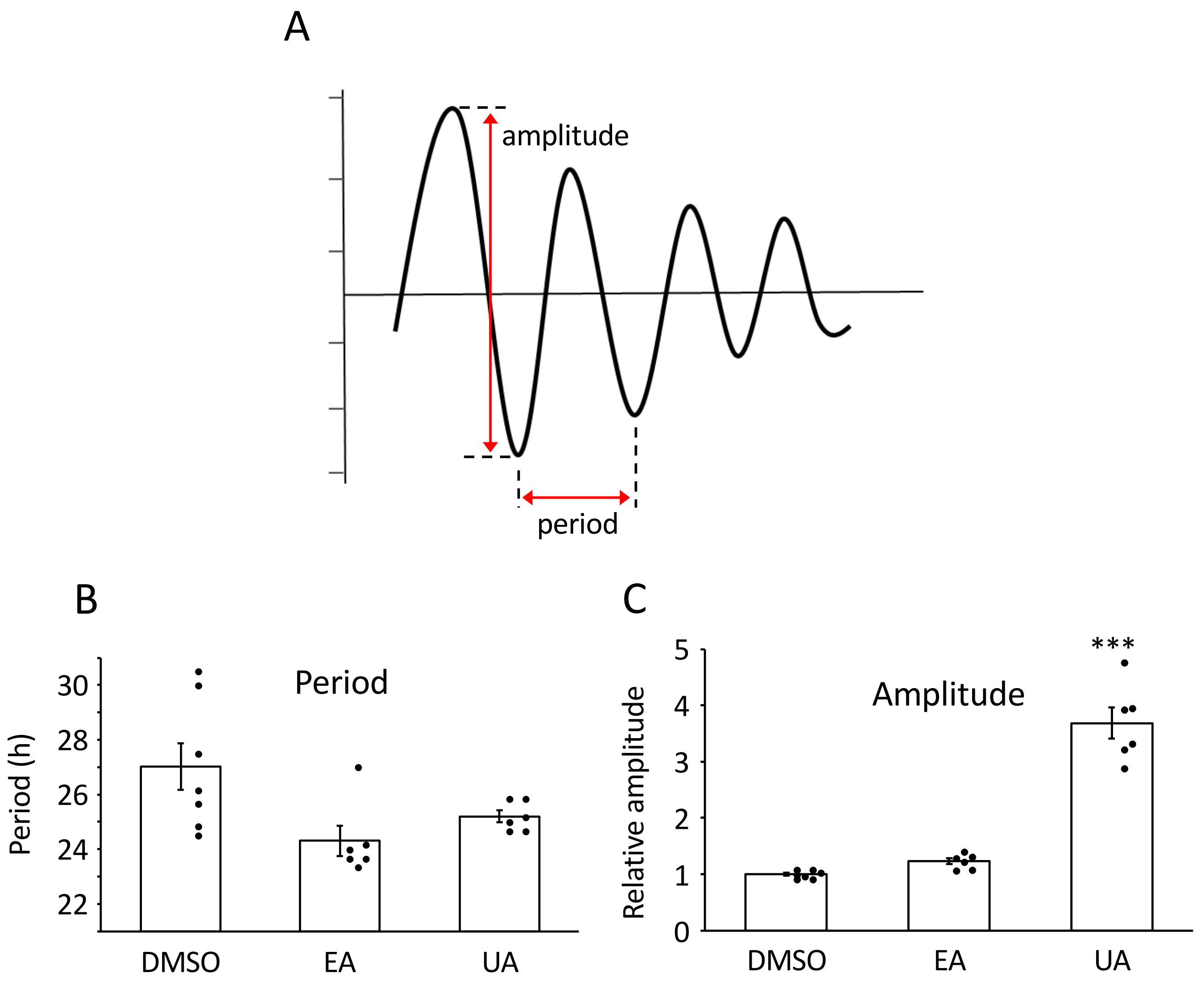

Effects of ellagic acid and urolithin A on the circadian clock of senescent cells
(A) The period was defined as the duration from first to second troughs. The amplitude was defined as the height from the first peak to the first trough.
(B, C) The period lengths and relative amplitudes were manually analyzed using the data from Fig.1A. Each sample number was 6 or 7. The value of DMSO was set to 1 for the relative amplitude. ANOVA followed by Dunnett's *post-hoc* test was analyzed. Statistical significance compared with the control "DMSO" is indicated as ***$p < 0.001$.
