# Supplementary material for: Urolithin A Modulates PER2 Degradation via SIRT1 and Enhances the Amplitude of Circadian Clocks in Human Senescent Cells
Source: Nutrients. 2024 Dec 25;17(1):20. doi: 10.3390/nu17010020 (PMC11722880; doi:10.3390/nu17010020)
Supplement: Supplementary file 1 [file nutrients-17-00020-s001.zip › SFig.3_R1.pdf]

SFig.3

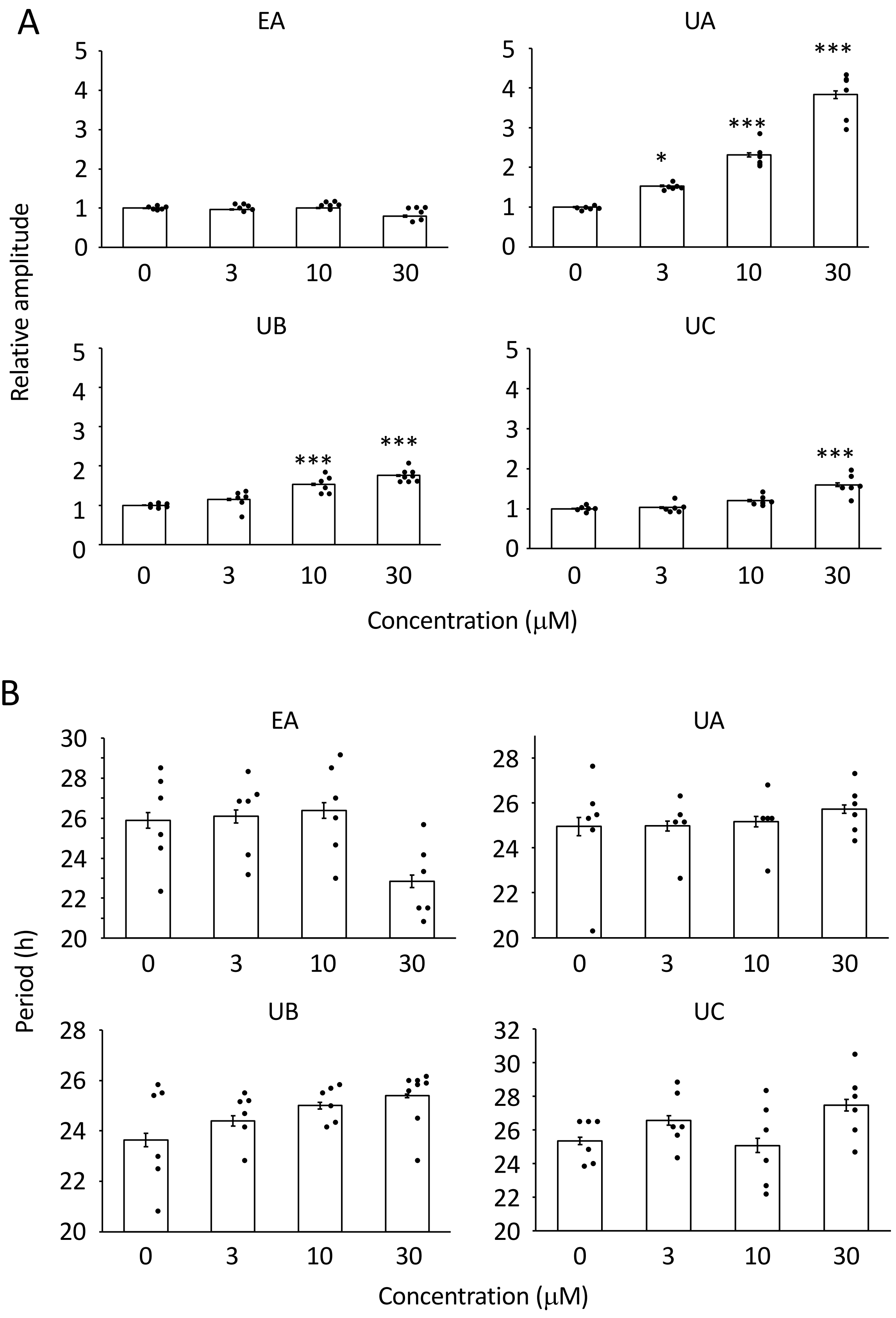

Effects of EA and its derivatives on the circadian clock of senescent cells

(A) Amplitudes were analyzed manually using the data from Fig. 2B and the amplitude of 0 for each metabolite was set to 1. (B) Period lengths were analyzed manually using the data from Fig. 2B. Each sample number was 5 or 6. Values are presented as the mean ± SEM. ANOVA followed by Dunnett's *post-hoc* test was analyzed. Statistical significance compared with the control "0" is indicated as **p* < 0.05, or ****p* < 0.001.
