# Supplementary material for: Urolithin A Modulates PER2 Degradation via SIRT1 and Enhances the Amplitude of Circadian Clocks in Human Senescent Cells
Source: Nutrients. 2024 Dec 25;17(1):20. doi: 10.3390/nu17010020 (PMC11722880; doi:10.3390/nu17010020)
Supplement: Supplementary file 1 [file nutrients-17-00020-s001.zip › SFig.4.pdf]

SFig.4

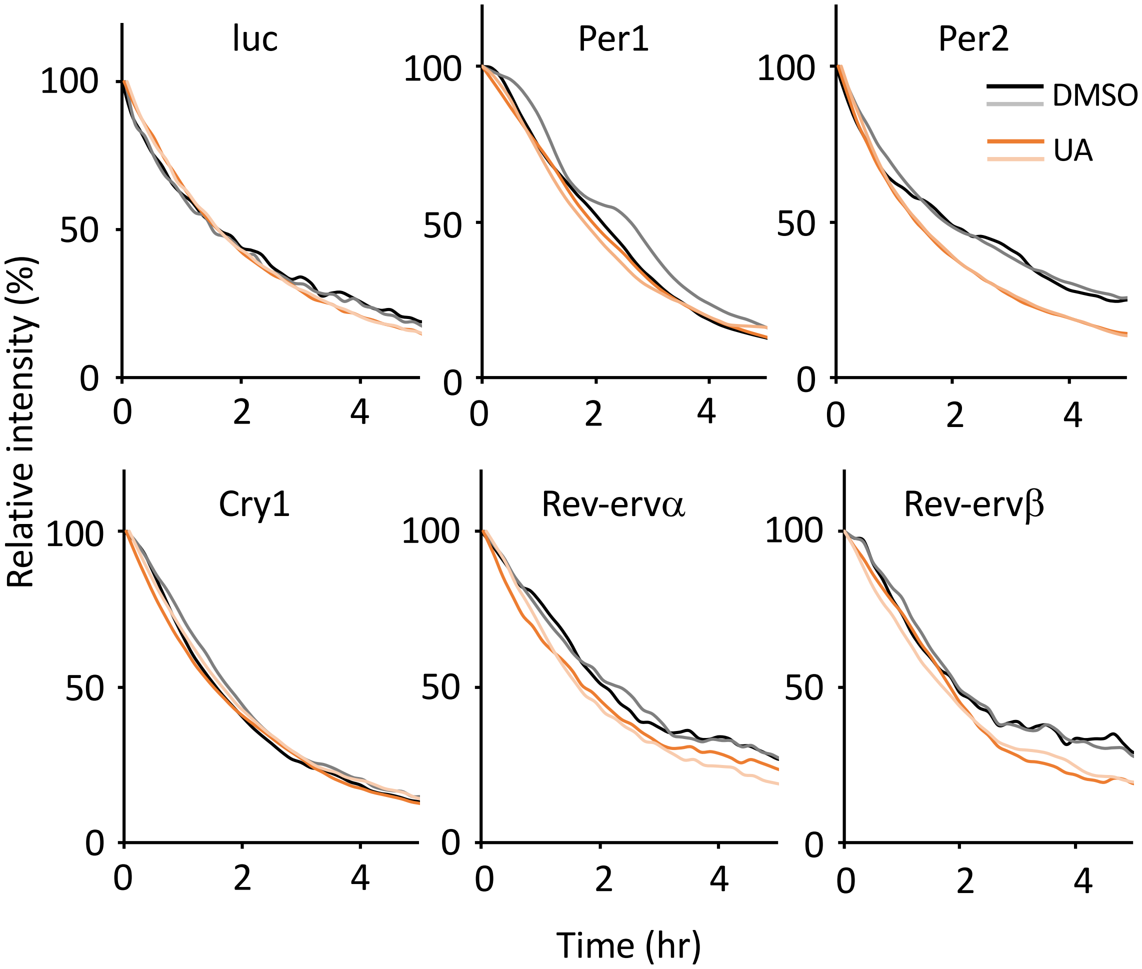

Effects of UA on the stability of circadian clock proteins in senescent cells
Luciferase-fused clock protein stabilities were measured using the real-time monitoring system.
