# Supplementary material for: Urolithin A Modulates PER2 Degradation via SIRT1 and Enhances the Amplitude of Circadian Clocks in Human Senescent Cells
Source: Nutrients. 2024 Dec 25;17(1):20. doi: 10.3390/nu17010020 (PMC11722880; doi:10.3390/nu17010020)
Supplement: Supplementary file 1 [file nutrients-17-00020-s001.zip › SFig.5_R1.pdf]

SFig.5

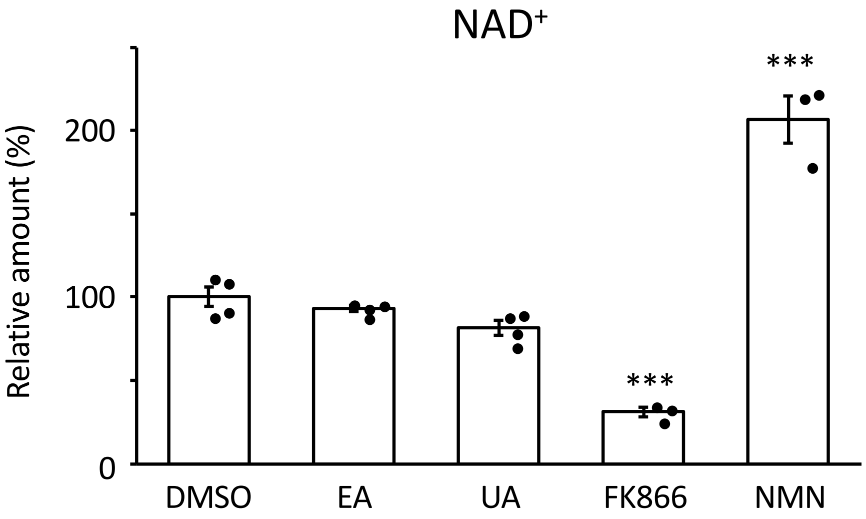

Effects of UA on NAD^+ amount in senescent cells

NAD^+ levels in Cells treated with indicated reagents were measured using the NAD/NADH-Glo assay kit. NAD^+ level in DMSO-treated cells was set to 100. Values are presented as the mean ± SEM. Sample numbers were 3 or 4 for each condition. ANOVA followed by Dunnett's *post-hoc* test was analyzed. Statistical significance compared with the control "DMSO" is indicated as ***$p < 0.001$.
